# Factors associated with health-related quality of life in gynaecologic cancer survivors with lower limb lymphedema: a cross-sectional study in Taiwan

**DOI:** 10.1186/s12905-023-02340-0

**Published:** 2023-04-28

**Authors:** Kuei-An Cho, Ming-Huei Cheng, Whei-Mei Shih, Shu-Ching Chen

**Affiliations:** 1 Department of Nursing, Linkou Chang Gung Memorial Hospital, Taoyuan, Taiwan; 2 Division of Reconstructive Microsurgery, Department of Plastic and Reconstructive Surgery, College of Medicine, Chang Gung Memorial Hospital, Chang Gung University, Taoyuan, Taiwan; 3 Section of Plastic Surgery, The University of Michigan, Ann Arbor, MI USA; 4 Center of Lymphedema Microsurgery, Chang Gung Memorial Hospital, Taoyuan, Taiwan; 5 Graduate Institute of Gerontology and Health Care Management, Chang Gung University of Science and Technology, Taoyuan, 333 Taiwan; 6 School of Nursing and Geriatric and Long-Term Care Research Center, College of Nursing, Chang Gung University of Science and Technology, 261, Wen-Hua 1st Road, Guishan, Taoyuan, 333 Taiwan; 7 Department of Radiation Oncology and Proton and Radiation Therapy Center, Linkou Chang Gung Memorial Hospital, Taoyuan, Taiwan; 8 School of Nursing, College of Medicine, Chang Gung University, Taoyuan, Taiwan

**Keywords:** Gynaecologic cancer, Lower limb lymphoedema, Cancer survivor, Patient reported outcome, Health-related quality of life

## Abstract

**Backgrounds:**

Gynaecological cancer survivors may develop lower limb lymphoedema after surgery, which negatively impacts quality of life. The purposes of this study were (1) to assess the levels of symptom distress, depression, body image, and health-related quality of life (HRQoL); (2) to recognize factors associated with HRQoL related in gynaecologic cancer survivors with lower limb lymphoedema.

**Methods:**

A cross-sectional study was conducted with convenience sampling of gynaecologic cancer survivors with lower limb lymphoedema. Gynaecologic cancer survivors were assessed for symptom distress, depression, body image, and HRQoL. Multiple regression analysis was conducted to recognize the factors associated with HRQoL. Independent-samples t*-*test was used to compare symptom distress, depression, body image, and HRQoL by grade of lymphoedema.

**Results:**

The most common distressing symptoms of lower limb lymphoedema were lower extremity oedema, lower extremity tightness, and lower extremity stiffness. Worse HRQoL was associated with more symptom distress, less satisfaction with body image, a high grade of lymphoedema, and a longer duration of lower limb lymphoedema. These factors explained 76.5% of the variance in HRQoL. Gynaecologic cancer survivors with late grade lymphoedema experienced lower HRQoL and higher levels of symptom distress, depression, and greater dissatisfaction with body image than those who had early grade lymphoedema.

**Conclusions:**

Symptom distress had the strongest association with overall HRQoL and with all individual domains of HRQoL, except mental function. These results suggest that educating gynaecologic cancer survivors to assess lower limb lymphoedema-related problems, providing symptom management, and guiding survivors in physical activity to relieve lower extremity discomfort can improve HRQoL.

## Background

Cancer is a leading cause of death worldwide; in the United States, it accounting for 609,360 deaths and an estimated 1.9 million new cases diagnosed in 2022 [1]. Uterine cancer, cervical cancer, and ovarian cancer are most common cancers of the in 2022 cancers [2]. In Taiwan, approximately 6,000 females are diagnosed with gynaecologic cancer each year and 17,00 die [3]. The most common treatments are radical surgery with lymphadenectomy and radiation therapy (RT), and surgery with concurrent chemoradiation therapy (CCRT) [4]. Because of the disruption of the lymphatic system, with reduced pelvic drainage and accretion of protein-rich lymph fluid in the lower extremities [5], patients with gynaecologic cancer may develop lower limb lymphoedema and experience swelling, puffiness, itching, tightness, heaviness, pain, skin changes, and infection [6–8]. These problems may cause physical inactivity, psychological distress, and dissatisfaction with appearance, resulting in a diminished quality of life [9–13].

Gynaecologic cancer survivors with lower limb lymphoedema commonly report symptoms in the lower extremities of swelling, numbness, tightness, heaviness, tenderness, and aching that cause distress and lower their health-related quality of life (HRQoL) [14–16]. HRQoL refers to an individual’s perceived wellbeing in the physical, mental, and social domains, which are related to disease or treatment [17, 18]. Patients with lower extremities ulceration may develop cellulitis as a consequence of bacterial invasion the subcutaneous tissues [19]. Research has revealed that leg ulcers patients experienced worse HRQoL due to more severity of wound [20], ulcer-related pain, signs of infection and inflammation [21], lower socioeconomic status, old age, and longer ulcer duration [22]. A recent study found that, after gynaecologic cancer surgery, patients scored the HRQoL domains of mobility and physical symptoms the lowest [12]. Reduced HRQoL as associated with younger age [12], obesity [23], postoperative RT [23], surgical procedure with combined pelvic lymphadenectomy and para-aortic lymphadenectomy [23], greater symptoms related to lower limb lymphoedema (e.g., heaviness, swelling, and numbness) [12], and higher psychological distress [10]. Gynaecologic cancer patients who had preoperative frailty [24], obesity [25, 26], smoking [25, 26], and treated with complexity surgery [25, 26] were more likely to have severe postoperative complications.

The International Classification of Functioning, Disability, and Health model of health and health states proposed by World Health Organization focuses on the consequences of disease-related health changes, which are affected by body function and structure, activities, participation factors, and environmental and personal contextual factors [27, 28]. Based on this model, we assume that gynaecologic cancer survivors with lower limb lymphoedema experienced the worse HRQoL [9–13], which is associated with advanced cancer stage [23], longer duration of lower limb lymphoedema [23], more severe lower limb lymphoedema [23], lower physical performance [10], more symptom distress [10], greater levels of depression [10], and dissatisfaction with body image [10].

Few studies have explored this issue, and most have presented risk factors [7, 27, 29], taken place in Western countries [10], been conducted to develop an instrument [14, 30], or only studied patients within 5 years of surgery [12]. There is a lack of research studying how lower limb lymphoedema affects HRQoL among gynaecologic cancer survivors. Therefore, the purposes of this study were (1) to explore the characteristics of symptom distress, depression, body image, and HRQoL; and (2) to determine the factors related to HRQoL in gynaecologic cancer survivors with lower limb lymphoedema.

## Methods

### Design and sample

We adopted a cross-sectional and correlational study of patient-reported outcome from September 2020 to May 2022. A convenience sampling of gynaecologic cancer survivors with lower limb lymphoedema were recruited from the plastic and reconstruction outpatient department of a 3,700-bed medical centre in northern Taiwan. The inclusion criteria were: (1) age ≥ 20 years; (2) diagnosis of gynaecologic cancer; (3) receipt of gynaecologic cancer surgery combined with RT, chemotherapy, or CCRT completed > 3 months ago; (4) definitive lower limb lymphoedema as determined by Indocyanine Green lymphography or lymphoscintigraphy. The exclusion criteria were: (1) acute or chronic psychiatric disease; (2) cognitive impairment; or (3) functional status < 60 on Karnofsky Performance Status Scale [31].

### Ethical considerations

This study was approved by the Institutional Review Board of Chang Gung Medical Foundation in Taiwan (Number: 202000803B0). All procedures were conducted following the Declaration of Helsinki. Survivors signed consent before study assessments.

### Data collection

Potential study participants were referred by their physician. Subjects were invited to participate in this study after a full explanation of the research objectives. Participants were asked to complete a set of self-reported questionnaires.

### Measures

#### Symptom Distress Scale (SDS)

Symptom distress was assessed using the Chinese-language version of the Symptom Distress Scale (SDS), developed by McCorkle and Young [32]. The SDS consists of 30 items, with responses scored on a Likert scale from 1 (no distress at all) to 5 (as much distress as possible). The Chinese version of the SDS has 8 additional items related to symptom distress due to lower limb lymphoedema in gynaecologic cancer survivors, based on a literature review [33]. The scale has been evaluated by lower limb lymphoedema experts and preliminarily tested in gynaecologic cancer survivors with lower limb lymphoedema, showing acceptable validity and reliability. In this study, the Cronbach’s alpha value for the SDS was 0.94.

### Profile of Mood States–Depression and Dejection Subscale (POMS–Depression and dejection subscale)

The Profile of Mood States–Depression and Dejection subscale was used to assess depression. It consists of 8 items and is scores on a Likert-type scale ranging from 0 (not at all) to 4 (extremely), with higher scores indicate greater depression [34]. For the present study, the Cronbach’s alpha was 0.89.

### Body Image Scale (BIS)

Symptoms or distress about body image were measured using the Chinese-language version of the Body Image Scale (BIS) [35], developed by Hopwood [36]. This 10-item scale assesses three aspects of body image: affective, behavioural, and cognitive. Each item is scored from 0 (not at all) to 3 (very much). The total possible score ranges from 0 to 30, with higher scores indicating greater symptoms of distress about body image. In the present study, the Cronbach α was 0.94.

### Lymphoedema Functioning, disability and Health Questionnaire for Lower Limb Lymphoedema (Lymph-ICF-LL)

The HRQoL was assessed using the Chinese-language version of the Lymphoedema Functioning, Disability and Health Questionnaire for Lower Limb Lymphoedema (Lymph-ICF-LL) [37], developed by Devoogdt et al. [38]. The Lymph-ICF-LL consists of 28 items measuring five domains: physical function (6 items), mental function (6 items), general tasks and household (3 items), mobility (7 items), and life and social life (6 items). Each item is scored on a scale of 0 (no problem) to 10 (very severe problem). Summed scores are converted into a 0-100 scale for each domain and for total scores. Higher scores indicate more severe problems. In this study, the Cronbach’s α value was 0.96.

### Cheng’s lymphedema grading system

Cheng’s Lymphedema Grading System was used to assess the grade of lymphoedema [39]. Lymphoedema is measured in five grades based on circumference differentiation, which is the circumference of the lesioned limb subtracted from the healthy limb and divided by the circumference of the healthy limb. Measurements are taken 10 cm above and below the elbow, 15 cm above and below the knee, and 10 cm above the ankle. Grading ranges from 0 to IV: Grade 0, reversible, circumference differentiation < 9%; Grade I, mild, circumference differentiation 10–19%; Grade II, moderate, circumference differentiation 20–29%; Grade III, severe, circumference differentiation 30–39%; and Grade IV, very severe, circumference differentiation > 40%. In Cheng’s Lymphedema Grading System classification, Grade 0 to Grade II is classified as early grade lymphoedema and Grade III to Grade IV as late grade lymphoedema [39]. The scale has been widely used and demonstrated to be reliable in lymphoedema grading studies [39, 40].

### Karnofsky Performance Status (KPS) index

The Karnofsky Performance Status (KPS) index was used to evaluate performance status. It is a single item instrument with an 11-point score ranging from normal function (100%) to expired (0%) [31].

### Demographic and clinical characteristics form

Demographic characteristics included age, type of occupation, employment after diagnosis, marital status, education level, religion, and annual family income. Clinical characteristics included gynaecologic cancer diagnosis, cancer stage, medical treatment, severity of lower limb lymphoedema, performance status, time from gynaecologic cancer surgery to lower limb lymphoedema onset (in years), and time since lower limb lymphoedema onset (in years).

### Statistical analysis

Data were analysed using SAS for Windows, version 9.1 (SAS Institute, Inc., Cary, NC). Descriptive statistics were used to explore demographic and clinical characteristics, symptom distress, depression, body image, and HRQoL. Multiple regression was used for factors associated with HRQoL. The independent variables included cancer stage (early vs. advanced), time experiencing lower limb lymphoedema, the severity of lower limb lymphoedema, performance status, symptom distress, depression, and body image. The independent-samples *t-*test was used to compare symptom distress, depression, body image, and HRQoL in survivors with early grade lymphoedema to those with late grade lymphoedema; survivors were adults (< 65 years old) and those who were old adults (≥ 65 years old) [41].

## Results

### Survivor characteristics

Of the 86 eligible gynaecologic cancer survivors with lower limb lymphedema approached, one survivor declined to participate because she had no interest. The response rate was 98.8%. The average age of partcipants was 64.22 (standard error [SE] = 1.11) years. Most were housewives (n = 53, 62.4%), unemployed after diagnosis (n = 60, 70.6%), married (n = 68, 80%), had an elementary school education (n = 30, 35.3%), held Buddhist/Taoist religious beliefs (n = 56, 65.9%), and had an average family annual income less than New Taiwan Dollars (NT$) 200,000 (US$6,663) (n = 43, 50.6%). The most common gynaecologic cancer diagnosis was cervical cancer (n = 37, 43.5%), followed by endometrial cancer (n = 35, 41.5%), and a majority were stage I at initiatal diagnosis (n = 42, 49.4%). Most received surgery only (n = 40, 47.1%), had bilateral pelvic lymph nodes removed (64.7%), had more than 15 lymph nodes removed (76.5%), and had 7% or greater difference in limb circumference (71.8%). The majority also had lower limb lymphoedema at Grade III (n = 29, 34.1%) and had adequate KPS scores (70 to100) (Table 1).

**Table 1:** Demographic and clinical characteristics of survivors (*N* = 85)

Variable	Number (%)	Mean (SE)	Range
Age (years)		64.22(1.11)	25–69
Adults (< 65)	37(43.5)		
Old adults (≥ 65)	48(56.5)		
Type of occupation			
Housewife	53(62.4)		
Unskilled/ semi-skilled worker	7(8.2)		
Skilled worker	4(4.7)		
Clerk, shop owner, farm owner	4(4.7)		
Semi-professional	3(3.5)		
Professional	2(2.4)		
Other	12(14.1)		
Employment after diagnosis			
Unemployed	60(70.6)		
Changed work	2(2.4)		
Return to work after discharge	18(21.2)		
Sick Leave	5(5.9)		
Marital status			
Married	68(80)		
Unmarried	17(20)		
Education level			
Literacy	4(4.7)		
Elementary	30(35.3)		
Junior high	14(16.5)		
Senior high	17(20.0)		
College and above	20(23.5)		
Religion			
None	25(29.4)		
Buddhism/ Taoism	56(65.9)		
Christianity/ Catholicism	3(3.5)		
Other	1(1.2)		
Family annual income (NT$)(US$)			
≦200,000(US$6,663)	43(50.6)		
210,000(US$6,996) ~ 500,000(US$16,658)	23(27.1)		
510,000(US$16,691) ~ 1,000,000(US$33,317)	11(12.9)		
1,100,000(US$36,648) ~ 1,500,000(US$49,975)	8(9.4)		
Gynaecologic cancer diagnosis			
Cervical cancer	37(43.5)		
Endometrial cancer	35(41.5)		
Ovarian cancer	9(10.6)		
Others	4(4.7)		
Cancer stage			
Carcinoma in situ	10(11.8)		
I	42(49.4)		
II	19(22.4)		
III	9(10.6)		
IV	5(5.9)		
Medical treatment			
Surgery	40(47.1)		
Surgery + RT	17(20.0)		
Surgery + CT	7(8.2)		
Surgery + CCRT	18(21.1)		
Surgery + CCRT + target therapy	2(2.4)		
Surgery + target therapy	1(1.2)		
Pelvic lymph nodes removed			
Unilateral	13(15.3)		
Bilateral	55(64.7)		
Bilateral with para-aortic	17(20.0)		
Lymph nodes removed			
< 15	20(23.5)		
≥ 15	65(76.5)		
Limb circumference difference			
< 7%	24(28.2)		
≥ 7%	61(71.8)		
Grade of lymphoedema (Cheng’s Lymphedema Grading System)			
0	5(5.9)		
I	18(21.1)		
II	18(21.2)		
III	29(34.1)		
IV	15(17.6)		
KPS score (level)		82.94(1.26)	60–100
90 to 100	59(69.4)		
80 to 90	6(7.1)		
70 to 80	6(7.1)		
60 to 70	14(16.5)		
Time since gynaecologic cancer surgery to lower limb lymphedema onset (year)		8.62(1.08)	
Time since lower limb lymphedema onset (year)		4.89(0.64)	
Time since gynaecologic cancer surgery (year)		13.50(1.28)	

Abbreviations:

SE, standard error mean

NT$, New Taiwan dollars

US$, United States dollars

RT, radiotherapy

CT, chemotherapy

RT, radiotherapy

CCRT, concurrent chemoradiation therapy

KPS, Karnofsky performance score

### Levels of outcome variables

The score for overall mean symptom distress was 1.76 (SE = 0.85); the top three items were “lower extremity oedema” (mean = 4.01, SE = 0.13), “lower extremity tightness” (mean = 3.46, SE = 0.15), and “lower extremity stiffness” (mean = 3.46, SE = 0.16). The mean score for depression was 13.59 (SE = 0.16). The mean score for body image was 1.03 (SE = 0.15). The mean HRQoL score was 37.15 (SE = 2.68). Mean scores for the subscales were: physical function, 42.17 (SE = 2.56); mental function, 28.04 (SE = 3.31); general tasks/household, 23.86 (SE = 3.09); mobility, 23.86 (SE = 3.14); and life/social life 23.86 (SE = 3.62) (Table 2).

**Table 2:** Levels of symptom distress, depression, body image, and health-related quality of life related to lower limb lymphoedema (*N* = 85)

Variable	Mean (SE)/N(%)	Range	Theoretical scoring range
Body Mass Index (BMI)			
Underweight (< 18.5)	3(3.5)		
Normal (18.5 ≤ BMI < 24)	38(44.7)		
Overweight (24 ≤ BMI < 27)	18(21.2)		
Obesity (≥ 27)	26(30.6)		
Symptom distress (SDS)	1.76(0.85)	1.06 − 2.84	1 − 5
Lower extremity edema	4.01(0.13)		
Lower extremity tightness	3.46(0.15)		
Lower extremity stiffness	3.46(0.16)		
Appearance change	3.21(0.16)		
Lower extremity skin redness	2.53(0.15)		
Lower extremity burning sensation	2.24(0.14)		
Lower extremity abnormal sensation	2.04(0.14)		
Depression (POMS–Depression and Dejection subscale)	13.59(1.15)	0 − 32	0 − 32
Body image (BIS)	1.03(0.09)	0–2.80	0–30
Lower limb lymphoedema related QOL (Lymph-ICF-LL)	37.15(2.68)	0.71–87.86	0–100
Physical function	42.17(2.56)		
Mental function	28.04(3.31)		
General tasks/household	23.49(3.09)		
Mobility	43.43(3.14)		
Life/social life	40.75(3.62)		
Abbreviations:			

BMI, Body Mass Index

SE, standard error mean

SDS, Symptom Distress Scale, higher scores reflect more symptom distress

POMS–Depression and Dejection subscale, Profile of Mood States–Depression and Dejection subscale

BIS, Body Image Scale, higher score indicating greater symptoms or distress about body image

Lymph-ICF-LL, Lymphoedema Functioning, Disability and Health Questionnaire for Lower Limb Lymphoedema, higher score indicating worse QOL.

### Factors associated with HRQoL

Multiple regression analysis identified factors that were significantly and independently associated with HRQoL and five domains of HRQoL. Gynaecologic cancer survivors who had greater symptom distress (β = 0.576), more dissatisfaction with body image (β = 0.345), a higher grade of lymphoedema (β = 0.141), or a longer time since lower limb lymphoedema onset (β = 0.116) were more likely to have worse overall HRQoL. These four factors explained 76.5% of the total variance in overall HRQoL. In terms of the subscales, physical function was lower in those who had more symptom distress (β = 0.728), and more dissatisfaction with body image (β = 0.185), which factors together explained 67.5% of the total variance in physical function. Greater limitation in mental function was associated with more dissatisfaction with body image (β = 0.523), a higher level of depression (β = 0.185), and early cancer stage (β = − 0.148), which together explained 67.2% of the total variance in mental function. General tasks and household scores were lower in survivors who had more symptom distress (β = 0.642), lower performance status (β = − 0.201), and longer time since lower limb lymphoedema onset (β = 0.178), which together explained 56.5% of the total variance in general tasks and household. Greater mobility limitation was associated with greater symptom distress (β = 0.643) and a higher grade of lower limb lymphoedema (β = 0.251), which together explained 56.5% of the total variance in mobility. Life and social life were worst in survivors who had more symptom distress (β = 0.511), higher grade lymphoedema (β = 0.254), and more dissatisfaction with body image (β = 0.234), which together explained 60.1% of the total variance in life and social life. Symptom distress was the factor most commonly associated with overall HRQoL and with all individual domains of HRQoL, except for mental function (Table 3).

**Table 3:** Factors significantly associated with overall health-related quality of life related to lower limb lymphoedema and five domains of health-related quality of life related to lower limb lymphoedema based on multiple regression analysis (*N* = 85)

Domains of health-related quality of life	Predictive Variable	Adjusted R^2^	Beta	F	p	95% CI	
						Lower	Upper
Overall health-related quality of life (Lymph-ICF-LL)	Symptom distress (SDS)	0.765	0.576	69.273	0.001	24.488	13.221
	Body image (BIS)		0.345		0.001	6.425	13.678
	Grade of lymphoedema		0.141		0.022	0.442	5.475
	Time since lower limb lymphoedema onset (year)		0.116		0.036	0.019	0.556
	Constant				0.001	−47.626	−26.018
Physical function (Lymph-ICF-LL)	Symptom distress (SDS)	0.675	0.728	88.364	0.001	30.220	44.342
	Body image (BIS)		0.185		0.009	1.299	8.983
	Constant				0.001	−42.528	−18.722
Mental function (Lymph-ICF-LL)	Body image (BIS)	0.672	0.523		0.001	11.814	25.872
	Depression (POMS–Depression and Dejection subscale)		0.371		0.001	4.101	13.007
	Cancer stage (early vs. advanced)		−0.148		0.023	−22.477	−1.729
	Constant						
General tasks/household (Lymph-ICF-LL)	Symptom distress (SDS)	0.565	0.642	37.427	0.001	30.525	48.819
	Performance status (KPS)		−0.201		0.010	−0.867	−0.120
	Time since lower limb lymphoedema onset (year)		0.178		0.019	0.087	0.932
	Constant				0.627	−49.006	29.719
Mobility (Lymph-ICF-LL)	Symptom distress (SDS)	0.565	0.643	55.478	0.001	30.932	49.858
	Grade of lymphoedema		0.251		0.001	2.466	9.896
	Constant				0.001	−58.856	−25.376
Life/social life (Lymph-ICF-LL)	Symptom distress (SDS)	0.601	0.511	143.259	0.001	2.831	15.575
	Grade of lymphoedema		0.254		0.001	25.808	48.194
	Body image (BIS)		0.234		0.005	2.887	11.555
	Constant				0.001	−69.620	−31.884

SDS, Symptom Distress Scale, higher scores reflect more symptom distress

BIS, Body Image Scale, higher score indicating greater symptoms or distress about body image

Lymph-ICF-LL, Lymphoedema Functioning, Disability and Health Questionnaire for Lower Limb Lymphoedema, higher score indicating worse QOL.

1. Dependent variable: health-related quality of life (Lymph-ICF-LL).

2. Input independent variable: covariates included cancer stage (early vs. advanced), time since lower limb lymphoedema onset (continuous score), grade of lymphedema, performance status (continuous score), symptom distress (continuous score), depression (continuous score), and body image (continuous score)

CI, confidence interval

### Differences in symptom distress, depression, body image, and HRQoL by grade of lymphoedema and age

Of the 85 participants, 41 were classified as having early grade lymphoedema and 44 were classified as having late grade lymphoedema. Independent-samples t-test was used to examine the differences in symptom distress, depression, body image, and HRQoL between the two groups. Compared to those who had early grade lymphoedema, participants who had late grade lymphoedema had statistically significantly worse scores for symptom distress, depression, body image, and HRQoL. Of the 85 participants, 33 were classified as adults and 45 were classified as old adults. Compared to those who were adults, participants who were old adults who had higher scores for symptom distress, body image, HRQoL, physical function, general tasks/household, mobility, and life/social life, but these differences were not statistically significant (Table 4).

**Table 4:** Differences in symptom distress, depression, body image, and health-related quality of life related to lower limb lymphoedema By grade of lymphoedema and age (*N* = 85)

Variables	Grade of lymphedema (Cheng’s Lymphedema Grading System)		*t*	*p* value	Age		*t*	*p* value
	Early (n = 41)	Late (n = 44)			Adults (n = 37)	Old adults (n = 48)		
	Mean (SE)	Mean (SE)			Mean (SE)	Mean (SE)		
Symptom distress (SDS)	1.59(0.05)	1.91(0.08)	−3.463	0.001	1.73(0.07)	1.78(0.07)	−0.457	0.649
Depression (POMS–Depression and Dejection subscale)	10.12(1.42)	16.82(1.65)	−3.055	0.003	13.46(1.81)	13.69(1.50)	−0.098	0.922
Body image (BIS)	0.66(0.11)	1.37(0.13)	−4.282	0.001	1.04(0.14)	1.02(0.12)	−0.077	0.939
Health-related quality of life (Lymph-ICF-LL)	24.65(3.27)	48.80(3.35)	−5.147	0.001	34.52(4.08)	39.18(3.55)	−0.863	0.391
Physical function	33.50(3.22)	50.27(3.54)	−3.485	0.001	39.91(4.10)	43.92(3.26)	−0.776	0.440
Mental function	16.22(4.09)	39.05(4.58)	−3.717	0.001	33.33(5.64)	23.96(3.89)	1.369	0.176
General tasks/household	13.28(3.74)	33.03(4.41)	−3.417	0.001	19.64(4.02)	26.46(4.50)	−1.096	0.276
Mobility	30.31(3.72)	55.65(4.25)	−4.461	0.001	37.88(4.25)	47.71(4.43)	−1.565	0.121
Life/social life	23.33(4.44)	56.97(4.43)	−5.358	0.001	33.83(5.38)	46.08(4.79)	−1.697	0.093

SE, standard error mean

SDS, Symptom Distress Scale, higher scores reflect more symptom distress

POMS–Depression and Dejection subscale, Profile of Mood States–Depression and Dejection subscale

BIS, Body Image Scale, higher score indicating greater symptoms or distress about body image

Lymph-ICF-LL, Lymphoedema Functioning, Disability and Health Questionnaire for Lower Limb Lymphoedema, higher score indicating worse QOL.

## Discussion

Our study identified the levels of symptom distress, depression, body image, and HRQoL and the factors impacting HRQoL in gynaecologic cancer survivors. Worse HRQoL outcomes were associated with more symptom distress, lower satisfaction with body image, higher grade lymphoedema, and a longer time since the onset of lower limb lymphoedema. Gynaecologic cancer survivors with lower limb lymphoedema in the present study reported the most distressing symptoms were lower extremity oedema, lower extremity tightness, lower extremity stiffness, appearance changes, lower extremity skin redness, lower extremity burning sensations, and abnormal lower extremity sensations. These findings support those of previous studies, which reported the most common stressors related to lower limb lymphoedema were swelling, numbness, tightness, heaviness, tenderness, and aching [14, 38]. All subjects in our study received gynaecologic cancer surgery with lymphadenectomy and had suffered from lower limb lymphoedema for a mean of 8.62 years after their surgery. Therefore, lower limb lymphoedema care involves lower extremity skin care, massage, and exercise. Clinicians caring for survivors of gynaecologic cancer should provide support and palliative symptom relief for lower limb lymphoedema.

Participants in the present study noted the most difficulty in HRQoL due to the following: “mobility,” “physical function,” “life and social life,” “mental function,” and “general tasks and household.” These results are similar to those of Hsu et al. [12], who found the worst HRQoL outcomes were associated with deficits in “mobility,” “physical function,” “general tasks and household,” “mental function,” and “life and social life.” However, subjects in our study reported higher levels of HRQoL than those reported by Hsu et al. [12]. The differences in HRQoL between studies may have been affected by treatment status and time since gynaecologic cancer surgery. Our study examined gynaecologic cancer survivors who had completed treatment. Hsu et al. [12] included women who had received gynaecologic cancer surgery, but 19.5% of them were still undergoing treatment. Participants in the study of Hsu et al. had surgery a mean of 2.5 years prior to lower limb lymphoedema onset, but the mean time from gynaecologic cancer surgery to lower limb lymphoedema onset in our subjects was 13.50 years. Lower limb lymphoedema may develop progressively after gynaecologic cancer surgery with lymphadenectomy. Early detection of lower limb lymphoedema after gynaecologic cancer surgery and educating survivors in self-monitoring can help ensure timely referral and treatment.

Results of the present study showed that gynaecologic cancer survivors with greater levels of symptom distress, lower satisfaction with body image, high grade lymphoedema, and a longer time since lower limb lymphoedema onset were more likely to report worse overall HRQoL. This finding supports those of previous studies [10, 12, 19, 20], which found that greater symptom distress increases the likelihood of physical dysfunction, psychological problems, and more concerns with body image, all of which can then lead to a decline in daily functioning. The findings suggest that healthcare providers should educate gynaecologic cancer survivors about assessing and managing the symptoms of their lower extremities. They should also monitor these survivors for any mental stress resulting from these symptoms, and encourage them to proactively improve their physical and mental health.

Our results indicate that symptom distress was the most common factor associated with overall HRQoL and most of its domains, except for “mental function.” These results differ from those of a previous study [12], which reported that fatigue was significantly associated with QoL. The differences between the two studies may be due differences in inclusion criteria. All gynaecologic cancer survivors in our study had completed treatment; by contrast, one fifth of subjects in the previous study [12] were actively receiving treatment and most had just completed the treatment period. Hence, improving fatigue and increasing physical fitness during and shortly after treatment will help gynaecologic cancer survivors cope with problems associated with lower limb lymphoedema and improve HRQoL.

Our results also found that gynaecologic cancer survivors with late grade lymphoedema perceived higher levels of symptom distress, more depression, a worse body image, and lower HRQoL than those who had early grade lymphoedema. More than half of our participants had bilateral pelvic lymph nodes removed and one-fifth of our participants had bilateral had bilateral pelvic lymph nodes removed; one-fifth also had para-aortic nodes removed. Furthermore, two thirds of our participants had more than 15 lymph nodes removed and nearly half received postoperative RT CCRT, which makes our population similar to those of previous studies [5] [23]. Survivors with late grade lymphoedema can prevent progression of lower limb lymphedema by elevating the lower extremities and by avoiding tight clothing, prolonged dropping, and prolonged exposure to sunlight or a high temperature.

D’Oria et al.’s [24] review reported that preoperative frailty was one of the major factors of adverse postoperative outcomes and overall survival. Gynaecologic cancer patients with preoperative frailty may increase the length of hospital stay, the risks of readmission and death postoperative. In our study, approximately one-third of survivors had required occasional assistance to minor signs and symptoms of disease of performance status (60–90 of KPS score) and those who 21.2% of survivors were overweight and 30.6% of survivors were obesity. However, symptoms of lower extremities cause restricted activities and decline in physical fitness. Health care providers should assess their health status, strength physical fitness, and prevent prefrailty and frailty.

We also found that the survivors who were old adults had higher scores for symptom distress, lower satisfaction with body image, worse HRQoL and five domains of HRQoL than those survivors were adults. Agreement between results of prior studies supports the assumption that old age patients reported more severe postoperative complications [25, 26]. The findings suggest that assessment and care are needed for both postoperative complications and impact on HRQoL.

### Limitations

This study had some limitations. First, participants’ pre-existing (pre-surgery) self-image of their lower extremities was not available and may affect the baseline HRQoL. Studies are needed to identify the correlation between satisfaction with body image and HRQoL. Second, we randomly selected gynaecologic cancer survivors with lower limb lymphedema from a plastic and reconstruction outpatient department of a medical centre in northern Taiwan. Most of our subjects intended to ask for surgery to address their lymphedema. Comparative studies are needed to examine the different motivations for surgery and its effects on lower limb lymphedema and HRQoL. Finally, the cross-sectional design of the study identified factors associated with HRQoL in gynaecologic cancer survivors. Lower limb lymphedema develops progressively after gynaecologic cancer surgery and the results may have limited interpretation. Longitudinal studies are needed to identify changes over time in HRQoL after the onset of lower limb lymphedema.

## Conclusions

We found the most common symptoms of distress in gynaecologic cancer survivors with lower limb lymphedema were lower extremity oedema, lower extremity tightness, and lower extremity stiffness. Survivors who reported worse HRQoL were also more likely to have greater symptom distress, greater dissatisfaction with body image, high grade lymphoedema, and a longer time since lower limb lymphedema onset.

### Clinical implications

Survivorship care of gynaecologic cancer patients with lower limb lymphedema after surgery should include patient education about lower limb lymphedema, symptom management, and physical activity to relieve lower extremity discomfort and improve HRQoL.

## Data Availability

The data that support the findings of this study are available from the corresponding author. Restrictions apply to the availability of these data, which were used under license for this study. Data are available from the authors with the permission of the Chang Gung Memorial Hospital Research Program (CMRP).

## Abbreviations

BIS: Body Image Scale
BMI: Body Mass Index
CCRT: concurrent chemoradiation therapy
CT: chemotherapy
HRQoL: health-related quality of life
ICF: International Classification of Functioning, Disability, and Health
ICG: Indocyanine Green
KPS: Karnofsky Performance Status
LLL: lower limb lymphoedema
Lymph-ICF-LL: Lymphoedema Functioning, Disability and Health Questionnaire for Lower Limb Lymphoedema
POMS: Profile of Mood States
RT: radiation therapy
SE: standard error mean
SDS: Symptom Distress Scale
WHO: World Health Organization
